# Reference Values for Birth Weight in Relation to Gestational Age in Poland and Comparison with the Global Percentile Standards

**DOI:** 10.3390/jcm12175736

**Published:** 2023-09-03

**Authors:** Agnieszka Genowska, Birute Strukcinskiene, Joanna Bochenko-Łuczyńska, Radosław Motkowski, Jacek Jamiołkowski, Paweł Abramowicz, Jerzy Konstantynowicz

**Affiliations:** 1 Department of Public Health, Medical University of Bialystok, 15-295 Bialystok, Poland; 2 Faculty of Health Sciences, Klaipeda University, LT-92294 Klaipeda, Lithuania; 3 Private Clinic of Obstetrics and Gynecology of Dr. J. Tomaszewski, 15-224 Bialystok, Poland; jluczynska@wp.pl; 4 Department of Pediatrics, Rheumatology, Immunology and Metabolic Bone Diseases, Medical University of Bialystok, University Children′s Hospital, 15-274 Bialystok, Poland; radek@umb.edu.pl (R.M.); pawel.abramowicz@umb.edu.pl (P.A.); jurekonstant@o2.pl (J.K.); 5 Department of Population Medicine and Lifestyle Diseases Prevention, Medical University of Bialystok, 15-269 Bialystok, Poland; jacek.jamiolkowski@umb.edu.pl

**Keywords:** percentiles, growth charts, birth weight, gestational age, newborns, singleton birth, Poland, INTERGROWTH-21, World Health Organization

## Abstract

Introduction. Percentiles of birth weight by gestational age (GA) are an essential tool for clinical assessment and initiating interventions to reduce health risks. Unfortunately, Poland lacks a reference chart for assessing newborn growth based on the national population. This study aimed to establish a national reference range for birth weight percentiles among newborns from singleton deliveries in Poland. Additionally, we sought to compare these percentile charts with the currently used international standards, INTERGROWTH-21 and WHO. Materials and Methods. All singleton live births (*n* = 3,745,239) reported in Poland between 2010 and 2019 were analyzed. Using the Lambda Mu Sigma (LMS) method, the Generalized Additive Models for Location Scale, and Shape (GAMLSS) package, smoothed percentile charts (3–97) covering GA from 23 to 42 weeks were constructed. Results. The mean birth weight of boys was 3453 ± 540 g, and this was higher compared with that of girls (3317 ± 509 g). At each gestational age, boys exhibited higher birth weights than girls. The weight range between the 10th and 90th percentiles was 1061 g for boys and 1016 g for girls. Notably, the birth weight of Polish newborns was higher compared to previously published international growth standards. Conclusion. The reference values for birth weight percentiles established in this study for Polish newborns differ from the global standards and are therefore useful for evaluating the growth of newborns within the national population. These findings hold clinical importance in identifying neonates requiring postbirth monitoring.

## 1. Introduction

Percentiles of birth weight are an essential tool for assessing neonatal health in both epidemiological research and clinical practice [1,2]. These percentile reference charts represent smoothed birth weight at gestational age (GA) and are used for clinical measurements. Small-for-gestational age (SGA) or large-for-gestational age (LGA), usually defined below the 10th or above the 90th percentile, approximates adverse newborn outcomes [3,4,5,6]. The etiology of fetal growth disturbances is multifactorial and may result from a number of cumulative mechanisms, including fetal, maternal, and placental factors. Epigenetic factors interfering with those previously mentioned are also involved, and may modify these processes [7]. Abnormal fetal weight gain has been associated with an increased risk of serious postpartum complications, such as newborn morbidity or mortality, and can have long-term impacts on future health. Studies have linked abnormal fetal weight gain to a higher risk of hypertension, obesity, metabolic syndrome, and type 2 diabetes in adulthood [8,9,10]. Consequently, the early identification of postnatal weight gain abnormalities using growth charts are essential, allowing for appropriate measures to be taken to achieve better health and growth outcomes for children. Several methods and practical approaches have been investigated within the last two decades for fetal growth assessment [11,12,13]. To increase the accuracy in the assessment of extreme fetal weights, an ultrasound estimation should be used [14].

Currently, the global birth weight standards INTERGROWTH-21 (IG-21) and the World Health Organization’s Fetal Growth Charts (WHO Fetal) are recommended for assessing birth weight [15,16,17]. These standards were established through rigorous methodologies in longitudinal observational cohort studies conducted in both low-income and high-income settings. The multicenter, multiethnic IG-21 project was implemented in 2009–2014 with the participation of 4321 pregnant women from eight geographical areas (Brazil, China, India, Italy, Kenya, Oman, United Kingdom, and the United States) [16]. Similarly, the WHO fetal study conducted between 2009 and 2015 included a population of 1387 pregnant women from 10 countries, representing various ethnic and cultural backgrounds (Argentina, Brazil, Democratic Republic of Congo, Denmark, Egypt, France, Germany, India, Norway, and Thailand) [17]. In both studies, only low-risk pregnant women with an accurate GA score of less than 14 weeks were recruited. The inclusion criteria ensured a healthy study population, considering individual characteristics such as age, height, body mass index, diet, smoking, and access to antenatal care, while excluding any socio-economic restrictions. The main difference in the inclusion criteria was the consideration of pregnancy complications and fetal factors, which were only included in the IG-21 study, resulting in similar birth weight values among the investigated countries [15,16,17]. Despite the active promotion of both IG-21 and WHO Fetal standards by many researchers [18,19,20,21], it remains unclear which chart should be preferred for clinical practice.

Distinguishing between normal and abnormal neonatal weight gain remains a significant challenge in obstetrics and pediatrics, not only in Poland but in many countries worldwide. The existing reference charts, such as IG-21 and WHO Fetal, do not include data from Poland, which is the largest country in Central−Eastern Europe, with an annual number of live births of approximately 350,000 [15,17,22]. Considering that there are ethnic differences in fetal growth around the world, the recommended growth curves may not fully reflect the weight gain patterns of Polish newborns, who exhibit a high degree of ethnic homogeneity. Moreover, in Poland, the reference charts for newborns based on the national population have not been updated for several decades. While some single-center authors have conducted studies over the last decade using local registry data from official hospital records [23,24], there is a need to establish current reference charts based on Polish population data to accurately identify suboptimal body weight in newborns.

This study aimed to establish a national reference range for birth weight percentiles among newborns from singleton deliveries in Poland. Additionally, we sought to compare these percentile charts with the currently used international standards IG-21 and WHO.

## 2. Materials and Methods

### 2.1. Study Design

In this study, we conducted a retrospective analysis of live births based on data from 3,849,388 individual birth records reported to the Central Statistical Office in Poland between 2010 and 2019. We used an anonymized data set to construct birth weight curves for singletons at GA between 23 and 42 weeks. These curves were then compared to the global birth weight standards of the IG-21 and the WHO [25,26]. Our results of birth weight curves for the population of Polish newborns were based on a complete dataset because, according to Polish legal regulations, each birth in the country is mandatorily registered. We obtained the data on live births from obstetric and neonatal medical records, which were consistently documented using a standardized uniform document throughout the country [22]. Trained medical personnel attending childbirth filled out individual birth cards and this information was subsequently transferred to the birth registry. The local Research Ethics Committee of the Medical University of Bialystok approved the study (approval number R-I-002/451/2018).

### 2.2. Newborn Parameters

Gestational age in our study was determined by the experienced obstetric staff and was based on the interval, measured in full weeks, between the first day of the last menstrual period (LMP) and the actual day of delivery. Additionally, birth weight was routinely measured immediately after birth using an electronic scale.

### 2.3. Exclusion Criteria and Study Population

**Figure 1:**
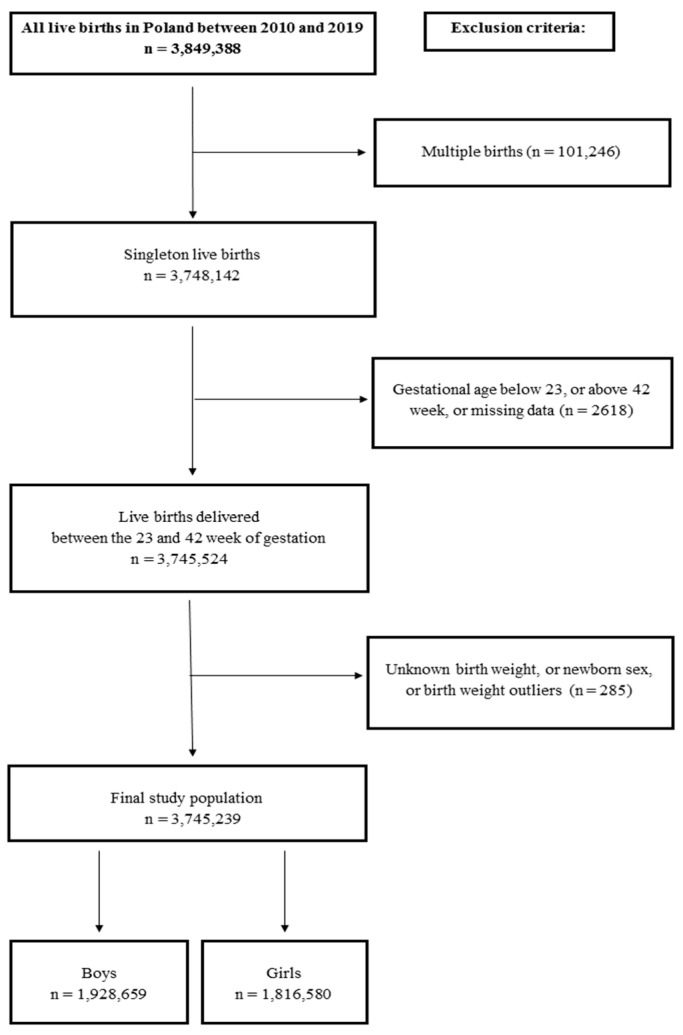
Flow chart of the study design.

To establish a normal range of birth weight, we applied certain exclusion criteria, which involved removing records of multiple births, GA below 23 weeks, and above 42 weeks, and cases of newborns without registered GA, birth weight, and sex of newborns, amounting to a total of 104,035 records. Additionally, using the Tukey method, we excluded 114 records containing outliers in birth weight [27]. After these exclusions, the study population consisted of 3,745,239 newborns, providing the essential data for estimating weight percentiles (Figure 1).

### 2.4. Statistical Analysis

In this study, we presented birth weight curves at 1-week intervals of gestation, focusing on single births. Live births between 23 and 42 weeks of gestation were classified into birth percentiles to establish a normal range of birth weight for GA. We created curves and tables for newborns, stratified by sex, using smoothed estimated curves, and for the mean and standard deviation (SD) calculated from the distribution of birth weights. Standards for calculating centiles SGA were defined as <3rd, <5th, and <10th percentiles, and LGA as >90th, >95th, >97th percentiles of birth weight for GA.

To achieve this, we utilized the Lambda−Mu−Sigma (LMS) method [28] to transform the data into sex-specific percentile curves. Various distribution models, such as Box−Cox Cole and Green, Box−Cox power exponential, and Box−Cox t (BCT), were tested. However, the BCT distribution, as described by Rigby and Stasinopoulos [29], provided the best fit and was selected for the final analysis presented in this paper. We employed the LMS method with the BCT distribution to estimate the percentile curves, which involved fitting age-specific curves for the mean, coefficient of variation, kurtosis, and skewness. These LMS parameters were estimated separately for boys and girls. To compare quantitative variables between groups of boys and girls, we used Student’s *t*-tests, while Pearson’s chi-square tests were used for qualitative variables. All calculations were performed using the R program. To fit the growth percentile curves, we employed the Generalized Additive Models for Location, Scale, and Shape (GAMLSS) package [30], and for visual representation, we used the ggplot2 package. As GAMLSS models were used, worm plots and detrended transformed Owen’s plots were also created to determine the goodness-of-fit and appropriateness of the calculations (Supplementary Material Figures S1 and S2). In the analysis, a *p*-value lower than 0.05 was considered statistically significant.

### 2.5. International Comparison

The sex-specific distribution of birth weight at GA in the Polish population between 2010 and 2019 was analyzed using the 3rd, 5th, 10th, 50th, 90th, 95th, and 97th percentiles. These percentiles were then compared to the global standards of IG-21 and the WHO [25,26].

## 3. Results

During the period from 2010 to 2019, the study included 1,928,659 boys and 1,816,580 girls from single pregnancies, resulting in a gender ratio of 1.06. After assessing birth weight, it was found that boys and girls with low birth weights (<2500 g) accounted for 3.97% and 4.77%, respectively. When considering GA, preterm births (<37 weeks) for boys and girls were 6.01% and 5.18%, respectively, while term births (37–41 weeks) were 92.63% and 93.40%.

The mean birth weight for boys was 3453 ± 540 g, which was 136 g higher than that of girls (3317 ± 509 g, *p* < 0.001). Notably, the difference in birth weight between boys and girls increased with GA, ranging from 22 g at 23 gestational weeks to 162 g at 41 and 42 gestational weeks, with statistical significance observed (*p* < 0.001) across the entire GA range (Table 1).

**Figure 2:**
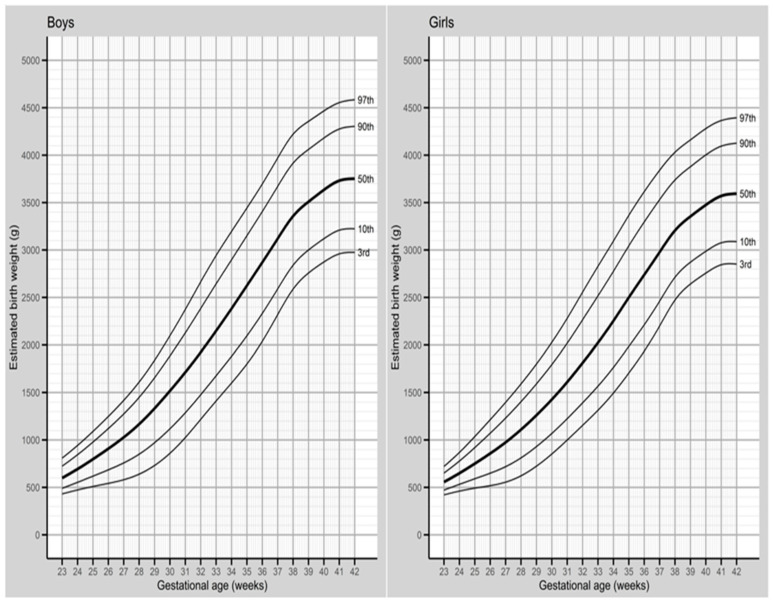
Normal range of birth weights according to gestational age among 365,003 singleton live births in Poland in the year 2019.

The normal range of birth weight, corrected for gestational weeks 23 to 42, for singleton births in the year 2019, is presented in Figure 2 (and Supplementary Materials, Tables S1 and S2), stratified by the sex of the newborn. The corrected curves exhibited a smooth and even pattern, with a sigmoid shape, indicating a monotonic growth over the course of GA, which became more curved after 38 weeks of gestation. Additionally, singleton births within the GA of 23–42 weeks showed that girls experienced the fastest weight gain between 29 and 31 weeks at the 3rd and 10th percentiles, while boys showed slightly later weight gain, between 30 and 32 weeks of gestation. For the 50th, 90th, and 97th percentiles, the fastest weight gain for both sexes occurred between 23 and 24 weeks of gestation (Figure 2 and Supplementary Materials, Tables S1 and S2).

In the analyzed percentiles (3rd, 10th, 50th, 90th, and 97th) for singleton births, boys consistently exhibited higher birth weights than girls at each GA. For instance, at the 50th percentile, boys had a mean birth weight of 3634 g, which was, on average, 158 g higher than that of girls (3476 g). The difference in birth weight between the sexes increased as the pregnancy progressed, with the smallest difference observed in the 3rd percentile (boys showing a higher weight than girls by 10 g at the 23rd week of pregnancy, and 124 g at the 42nd week of pregnancy). At the 97th percentile, the differences were most pronounced, with an 88 g gap at 23 weeks of gestation and a 190 g gap at 42 weeks of gestation (Supplementary Materials, Tables S1 and S2).

### Comparison with the Global References

For babies born at 40 weeks of gestation in the Polish population, the birth weight values at the 3rd, 5th, 10th, 50th, 90th, 95th, and 97th percentiles were higher compared to both the IG-21 and the WHO standards. Between 2010 and 2015, which coincides with the IG-21 and WHO studies, boys born at 40 weeks of gestation in Poland had a birth weight of 3.60 kg at the 50th percentile, which was 0.22 kg higher than the IG-21 standard and 0.08 kg higher than the WHO standard. In girls, the birth weight at the 50th percentile was 3.44 kg, which was 0.18 kg higher than the IG-21 standard and 0.11 kg higher than the WHO standard (Table 2).

Over the decade, the birth weight among Polish newborns increased for both sexes, and weight gain in 2019 vs. 2010 was observed in all analyzed percentiles, except for LGA_97th_ in boys. The most significant weight gain occurred in the SGA_3rd–10th_ percentiles, with the lowest increase seen in the SGA_3rd_ percentiles for both boys (81 g) and girls (85 g) compared to SGA_5th_ (74 and 77 g, respectively) and SGA_10th_ (65 and 67 g, respectively). The weight gain for the 50th percentile was about half that of SGA_3rd_ (39 g for boys and 45 g for girls). In the LGA_90th–97th_ percentiles, the growth was slower compared to the other percentiles (Table 2).

**Figure 3:**
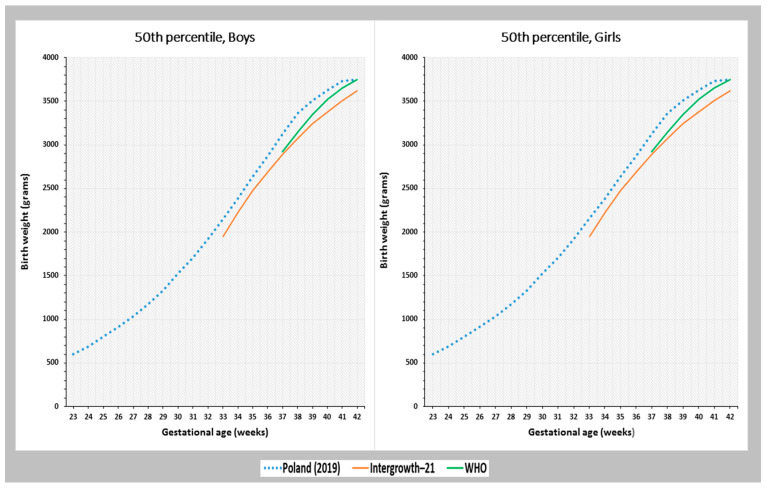
Birth weight at the 50th percentile based on gestational age among Polish newborns from singleton pregnancies, in comparison with the global references INTERGROWTH-21 and WHO.

Figure 3 shows the smoothed birth weight percentile curves for singleton births in the Polish population during 23–42 gestational weeks, compared to global references. As the IG-21 standard covers 33–42 weeks of gestation and the WHO standard covers 37–42 weeks of gestation, the birth weight percentiles were compared using these references. For the 50th percentile, the curve representing the Polish population showed a higher birth weight values for boys and girls in comparison to the IG-21 curves. These differences ranged from 130 g at 42 weeks of gestation to 290 g at 38 weeks of gestation for boys, and from 120 g at 34–35 weeks of gestation to 240 g at 38 weeks of gestation for girls.

Compared to the WHO standard, the birth weight at the 50th percentile among the Polish population was higher during the GA of 37–41 weeks. The minimum difference was observed in the 41st week of pregnancy, with boys weighing 80 g more and girls weighing 90 g more than the WHO standard. The maximum difference was seen in the 38th week of pregnancy, with boys weighing 210 g more and girls weighing 220 g more than the WHO standard. However, in the 42nd week of pregnancy, the birth weight values for both sexes in the Polish population were comparable to the WHO standard (Figure 3).

Values of percentile for SGA_3rd–10th_ and LGA_90th–97th_ in the population of Polish newborns were generally higher when compared to the IG-21 and the WHO standards. The exceptions were the results for SGA_3rd_ among girls born prematurely, which were similar when compared with the IG-21 standard. In addition, the values for LGA_90th–97th_ among boys born at 42 weeks of gestation were lower when compared to the WHO standard (Supplementary Material, Figure S3).

## 4. Discussion

### 4.1. Main Findings of the Study

In this study, we established reference values for birth weight according to the GA of 23–42 weeks, categorized by the sex of newborns in the Polish population. In the year 2019, at 40 gestational weeks, the birth weight at the 50th percentile for boys was 3634 g, which was, on average, 158 g higher than that of girls (3476 g). The range between the 10th and 90th percentiles was 1061 g for boys and 1016 g for girls. Over the analyzed decade, the values of birth weight from SGA_3rd_ to LGA_97th_ for newborns born at 40 weeks were consistently higher in the Polish population compared to the current IG-21 and the WHO global standards.

### 4.2. Interpretation of Results

Our results indicate that, compared to the global IG-21 standard, the birth weight of Polish newborns born at 40 weeks of gestation (in period 2010–2015) was higher for boys in SGA_3rd_ and SGA_10th_ by 190 g, and for girls by 150 g and 160 g, respectively, and for boys in LGA_90th_ and LGA_97th_ by 230 g and 240 g, and for girls by 180 g and 190 g, respectively. Similarly, when compared to the WHO standard, the birth weight for Polish newborns at 40 weeks was higher for both sexes in SGA_5th_ by 40 g, and in LGA_95th_ by 90 g. These discrepancies lead us to believe that the global standards may not be adequately adapted to the Polish context, and other authors have also raised doubts regarding the discrepancies in birth weight values [31,32,33]. Hence, we suggest caution in using IG-21 and WHO standards universally across all populations, as they may result in misclassifications of cut-off points. While the IG-21 and the WHO standards provide valuable information on optimal newborn weight, selecting a national reference for newborn size is essential to avoid diagnostic errors.

The percentile birth weight of Polish newborns was consistently higher when compared to the IG-21 and the WHO standards for low-risk populations, although our population was not entirely ideal. We included less strict criteria to reduce high-risk pregnancies, but this approach did not eliminate the maternal and fetal health burden. One possible explanation for these differences is the existence of geographical and regional variations in genetic characteristics, maternal phenotype, and physiology that can influence the course of pregnancy and fetal growth [34]. Furthermore, some studies have highlighted the impact of ethnic differences on physiological pregnancy characteristics and neonatal outcomes [35,36,37].

It is essential to emphasize that the Polish population is ethnically homogeneous, and this factor could have played an important role in the observed differences in birth weight when compared to Poland and the multiethnic populations used in the IG-21 and the WHO standards. Additionally, our analysis covered the stable period of 2010–2019, which is suitable for assessing weight percentiles due to stable health conditions. During this time, there was no significant mass influx of refugees from neighboring countries, which could introduce variations in health characteristics among pregnant women.

We also believe that the higher weight percentile values of Polish newborns may be attributed to specific factors such as socio-economic status, environment, and medical care, which can influence the health conditions of women of reproductive age and pregnant women. These conditions are generally related to the level of economic development in different countries. This is evident in the visible differences in newborn birth weights among high-income countries, such as Sweden (3623 g), the Netherlands (3542 g), Ireland (3514 g), and the USA (3502 g), compared to low-income countries, such as Bhutan (3210 g) and India (3055 g) [31].

Moreover, there is evidence that geographical settings may play a role in the similarity of birth weights among neighboring countries. For example, a study in Germany [38] reported averaged birth weights at the 50th percentile ranging from 595 to 3780 g in boys and from 570 to 3620 g in girls during the GA of 23–42 weeks, which were quite similar to our results in the Polish population. Our findings showed that for a GA of 23–42 weeks at the 50th percentile, the average birth weight in boys ranged from 599 to 3751 g, and that in girls ranged from 557 to 3595 g. These minor differences indicate that national populations exhibit specific characteristics for neonatal weight-for-gestational age, and to better identify neonates with suboptimal growth, it is important to use intervention thresholds developed specifically for the particular population.

An analysis of the 10-year changes in percentile birth weight in Poland showed an increase in median birth weight in 2019 compared to 2010. For boys, the median birth weight increased by 39 g, from 3634 to 3595 g, and for girls, it increased by 45 g, from 3476 to 3431 g. These results are consistent with other studies conducted in high-income countries, which also reported similar upward trends in birth weight since the end of the last century [39,40,41]. The observed increase in birth weight can be attributed mainly to lifestyle factors in women of reproductive age and pregnant women. For instance, the increasing rates of obesity have been associated with this trend [41,42]. Elevated body mass index levels in pregnant women increase the risk of common pregnancy complications, such as gestational diabetes and LGA infants. The role of obesity may be explained by fetal overfeeding due to increased nutrient transport across the placenta, leading to higher insulin synthesis and fetal growth [43,44]. It is crucial to emphasize that LGA is associated with an increased risk of interventions during delivery, such as cesarean sections and postpartum hemorrhage for the mother, and can also lead to serious health consequences for the fetus, such as perinatal mortality and, in later life, an increased risk of cancer, obesity, and type 2 diabetes [45,46]. Another possible factor associated with the increasing trend in birth weight is the reduction in smoking tobacco products [47]. However, assessing this impact can be challenging because a reduction in smoking during pregnancy occurred at a time when the rate of preterm births was increasing (from 6.55% to 7.41% during the period of 2010–2019), and the effects of these two variables may have been balanced out. This topic is of interest and might be included in future research agendas.

### 4.3. Strengths and Limitation

This is the first study to present birth weight percentiles according to GA for single births based on the entire Polish population. The use of a national registry of all live births, encompassing 3.7 million births, is a significant strength of this study, as it allowed for the analysis of birth weight for GA among preterm infants, which might be underrepresented in other studies [48]. Furthermore, the study examined weight percentile changes over a 10-year period from 2010 to 2019, which is a rare analysis in other countries [19,49]. The comprehensive and large dataset, covering the complete neonatal population, provided reliable reference ranges for estimating neonatal weight-for-GA in both sexes. Moreover, the advantage of the study lies in the small percentage of excluded and missing data (2.7%). This information can be invaluable for clinicians in identifying neonates who may require closer monitoring due to potential suboptimal growth. Additionally, the reference ranges established in this study can be helpful for epidemiologists tracking birth weight differences by geographic location and for use in high-income countries with similar demographic characteristics.

However, it is important to consider certain limitations when interpreting the study’s findings. The retrospective nature of the data used for the analysis limited the ability to examine factors related to adverse birth weight outcomes. For example, maternal chronic diseases like hypertension and gestational diabetes mellitus, which can affect fetal growth and the duration of pregnancy, were not included in the analysis [50]. It is possible that other undiagnosed factors, such as congenital defects, could have influenced newborn outcomes as well [51]. Additionally, maternal lifestyle data, including smoking and body weight, were missing from the birth records, despite their potential relevance [42,52]. Meta-analyses on pregnancy and birth cohorts, including the Polish population, have highlighted the association of continued smoking during pregnancy with a higher risk of SGA at birth (OR 2.15, 95% CI 2.07–2.23), as well as the association of maternal prepregnancy underweight with SGA (OR 1.67, 95% CI 1.58–1.76), and the association of prepregnancy obesity with LGA at birth (OR 2.28, 95% CI 2.19–2.37) [42,52].

Our study may have certain limitations related to the methodology used for collecting information on GA. It is important to note that the GA data relied on available information from official records in obstetric care, and the assessment of GA was conducted using either the LMP method or ultrasound. Therefore, the results should be interpreted with caution. It is worth mentioning that similar challenges in GA assessment have been encountered in other research studies [53,54]. To avoid the risk of overestimating the results, we also used Tukey’s method, a widely used approach in other studies [33], to detect and handle outliers.

An additional difficulty in interpreting our findings is the variations in the SGA and LGA cut-off values among different studies. The IG-21 study describes the 3rd, 10th, 50th, 90th, and 97th percentiles, while the WHO study employs the 5th, 50th, 90th, and 95th percentiles.

Despite these acknowledged limitations, we firmly believe that our current study, based on a large sample size, serves as a valuable Polish reference that can enhance the assessment of newborns and be beneficial to clinicians in their practice.

## 5. Conclusions

In this article, we have established a comprehensive reference of percentiles and curves for birth weight, utilizing a large population-based dataset of newborns from singleton deliveries in Poland over the period 2010–2019. Our research demonstrates that the birth weight of Polish neonates surpasses the global standards set by the IG-21 and the WHO, especially when compared to populations from countries with differing levels of economic development to Poland.

The findings of this study hold clinical significance as they can aid in the identification of newborns who require close monitoring during the immediate postnatal period. Additionally, they offer an opportunity to proactively prevent short-term and long-term adverse health outcomes. The reference values described by us can be used as a basis for testing in various SGA or LGA risk groups.

## Figures and Tables

**Table 1:** Normal range of birth weights according to gestational age in 3,745,239 singleton live births in Poland over the years 2010–2019.

GA at Delivery [Weeks]	Boys			Girls			Mean of Sex Differences [Grams]	*p*
	*n* = 1,928,659	Mean [Grams]	SD [Grams]	*n* = 1,816,580	Mean [Grams]	SD [Grams]		
23	615	603	85	529	581	82	22	<0.001
24	918	700	122	707	650	105	50	<0.001
25	1013	788	153	868	749	144	39	<0.001
26	1185	894	177	1054	853	198	41	<0.001
27	1496	1015	235	1233	960	238	55	<0.001
28	1860	1155	257	1500	1103	266	52	<0.001
29	2100	1310	286	1707	1230	292	80	<0.001
30	2871	1506	341	2439	1420	357	87	<0.001
31	3682	1687	367	2878	1601	370	86	<0.001
32	5602	1919	418	4391	1837	442	82	<0.001
33	7662	2137	409	6060	2037	430	100	<0.001
34	13,364	2378	427	10,649	2267	433	111	<0.001
35	23,633	2621	440	18,872	2508	438	113	<0.001
36	49,885	2867	441	41,149	2744	439	123	<0.001
37	119,346	3124	442	101,131	2987	433	137	<0.001
38	329,249	3348	432	296,488	3201	414	147	<0.001
39	540,203	3501	427	510,517	3348	408	153	<0.001
40	566,588	3622	432	558,599	3465	414	157	<0.001
41	231,119	3714	433	230,026	3552	414	162	<0.001
42	26,268	3751	450	25,783	3589	431	162	<0.001
Mean for 23–42 weeks		3453	540		3317	509	136	<0.001

Abbreviations: GA—gestational age, SD—standard deviation.

**Table 2:** Comparison of percentiles at 40 weeks of gestation, categorized by sex of the newborns from singleton pregnancies, between the Polish reference, the INTERGROWTH-21, and the WHO.

	n *	Smoothed Percentiles [Kilograms]						
		3	5	10	50	90	95	97
Boys								
INTERGROWTH–21 (years 2009–2014)	n = 2568	2.63		2.88	3.38	3.94		4.22
WHO (years 2009–2015)	n = 691		2.880		3.519	4.067	4.251	
Poland								
2010	n = 208,768	2.793	2.898	3.055	3.595	4.165	4.344	4.467
2011	n = 194,830	2.806	2.909	3.063	3.596	4.162	4.339	4.461
2012	n = 193,474	2.817	2.918	3.072	3.604	4.170	4.347	4.469
2013	n = 184,995	2.814	2.915	3.068	3.600	4.165	4.341	4.461
2014	n = 188,082	2.828	2.929	3.082	3.606	4.162	4.337	4.457
2015	n = 184,009	2.837	2.938	3.089	3.612	4.169	4.343	4.462
2016	n = 191,066	2.857	2.956	3.106	3.625	4.179	4.351	4.469
2017	n = 200,957	2.864	2.963	3.112	3.629	4.180	4.352	4.469
2018	n = 194,786	2.870	2.966	3.111	3.625	4.178	4.351	4.468
2019	n = 187,692	2.874	2.972	3.120	3.634	4.181	4.351	4.467
Change between 2010 and 2019 [grams]		+81	+74	+65	+39	+16	+7	0
Girls								
INTERGROWTH–21 (years 2009–2014)	n = 2523	2.55		2.78	3.26	3.80		4.08
WHO (years 2009–2015)	n = 608		2.748		3.336	3.871	4.060	
Poland								
2010	n = 193,294	2.672	2.771	2.920	3.431	3.976	4.150	4.269
2011	n = 183,307	2.681	2.780	2.928	3.438	3.980	4.152	4.270
2012	n = 182,545	2.694	2.791	2.937	3.446	3.990	4.160	4.277
2013	n = 174,647	2.696	2.792	2.937	3.441	3.982	4.152	4.268
2014	n = 177,248	2.709	2.803	2.945	3.446	3.984	4.152	4.267
2015	n = 174,136	2.721	2.815	2.957	3.454	3.986	4.153	4.267
2016	n = 180,833	2.734	2.828	2.971	3.469	4.002	4.168	4.282
2017	n = 190,079	2.745	2.837	2.977	3.472	4.007	4.176	4.292
2018	n = 183,180	2.752	2.843	2.983	3.473	4.004	4.171	4.286
2019	n = 177,311	2.757	2.848	2.987	3.476	4.003	4.168	4.280
Change between 2010 and 2019 [grams]		+85	+77	+67	+45	+27	+18	+11

* The year-specific sample size was the following for boys and girls, respectively.

## Data Availability

Data were collected from public datasets analyzed or generated during the study and presented in Table 1.

## Supplementary Materials

The following are available online at https://www.mdpi.com/article/10.3390/jcm12175736/s1. Figure S1. Worm plot for the assessment of goodness-to-fit in the data for boys and girls (left and right panel respectively). Figure S2. Detrended transformed Owen’s plot for boys and girls. Figure S3. Birth weight at the 3rd, 5th, 10th, 90th, 95th, 97th percentiles based on gestational age among Polish newborns from singleton pregnancies, in comparison with the global references INTERGROWTH-21 and WHO. Table S1. Normal range of birth weights according to gestational age in singleton live births for boys in Poland (2019). Table S2. Normal range of birth weights according to gestational age in singleton live births for girls in Poland (2019).
