# Supplementary material for: Reference Values for Birth Weight in Relation to Gestational Age in Poland and Comparison with the Global Percentile Standards
Source: J Clin Med. 2023 Sep 3;12(17):5736. doi: 10.3390/jcm12175736 (PMC10488537; doi:10.3390/jcm12175736)
Supplement: Supplementary file 1 [file jcm-12-05736-s001.zip › jcm-12-05736-s001.pdf]

Journal of
Clinical Medicine

Supplementary Materials

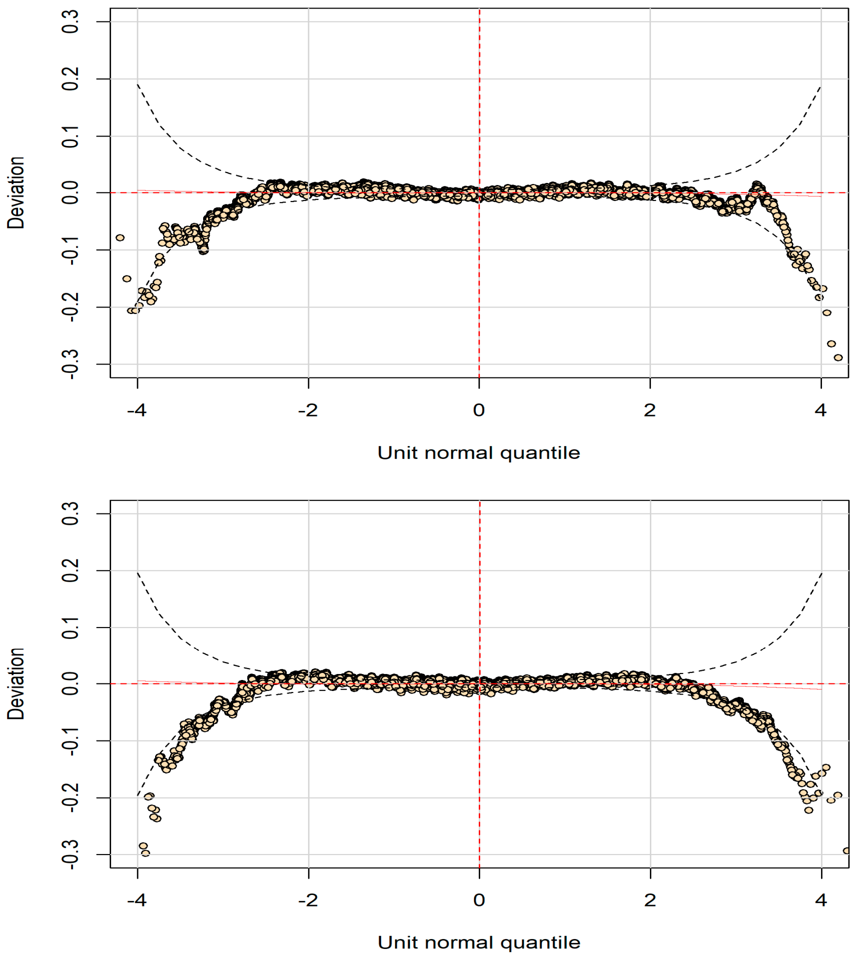

Figure S1. Worm plot for the assessment of goodness-to-fit in the data for **boys and girls** (upper and lower panel respectively)

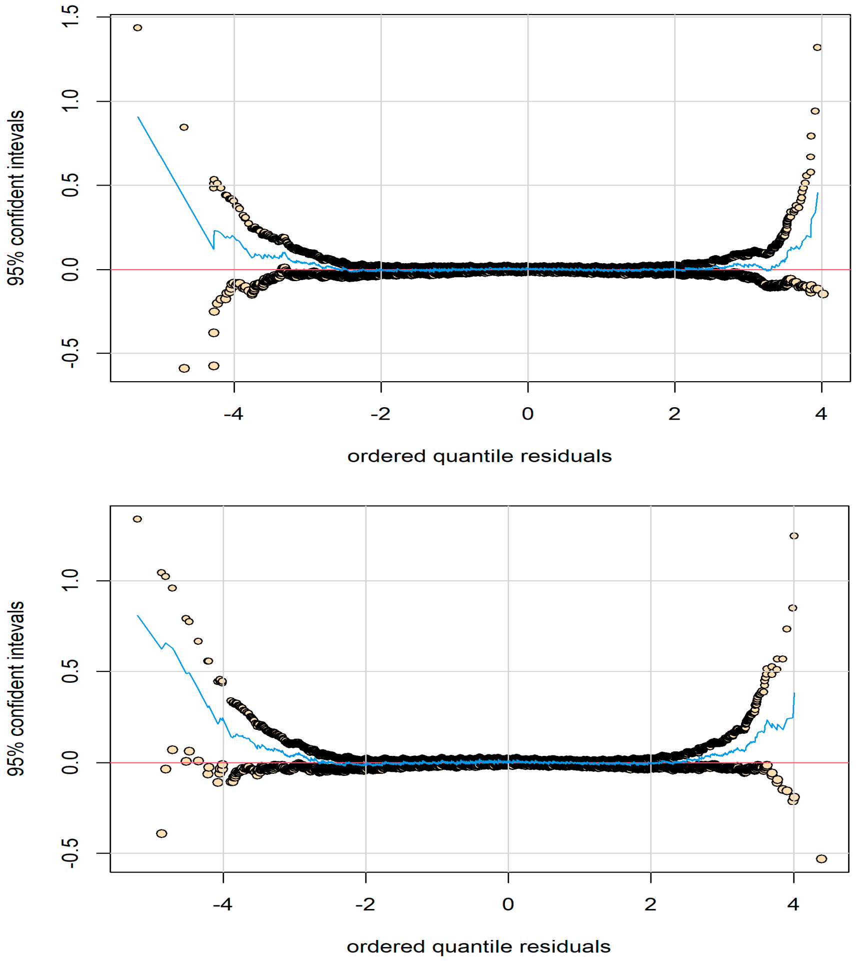

Figure S2. Detrended transformed Owen's plot for **boys and girls** (upper and lower panel respectively)

Table S1. Normal range of birth weights according to gestational age in singleton live births for **boys** in Poland (2019)

GA at delivery [weeks]	n= 187,692	Birth weight [grams]								
		Smoothed centiles							Mean	SD
		C3	C5	C10	C50	C90	C95	C97		
23	53	431	456	490	599	724	772	809	599	81
24	82	473	507	553	694	845	901	943	704	143
25	96	510	556	619	798	978	1042	1090	801	142
26	100	543	604	685	908	1121	1194	1248	911	164
27	154	581	657	758	1028	1275	1358	1418	1021	251
28	179	639	730	850	1167	1449	1543	1610	1136	238
29	204	731	834	971	1333	1655	1762	1839	1310	289
30	261	860	970	1118	1517	1885	2009	2098	1544	293
31	379	1025	1136	1287	1713	2128	2271	2375	1679	356
32	549	1218	1326	1477	1924	2385	2547	2665	1949	384
33	808	1411	1521	1676	2149	2644	2817	2943	2152	417
34	1351	1600	1716	1881	2383	2897	3071	3196	2392	404
35	2340	1800	1923	2097	2626	3151	3323	3443	2620	441
36	4915	2039	2160	2332	2870	3406	3578	3697	2872	433
37	12,214	2320	2430	2591	3121	3671	3848	3971	3125	438
38	35,808	2589	2689	2840	3357	3916	4096	4221	3372	432
39	57,841	2758	2855	3001	3509	4060	4236	4356	3522	421
40	51,154	2874	2972	3120	3634	4181	4351	4467	3644	423
41	18,260	2960	3059	3209	3731	4276	4442	4554	3739	419
42	944	2976	3074	3225	3751	4304	4472	4584	3754	449

Abbreviations: C—Centile, GA—gestational age, SD—standard deviation

Table S2. Normal range of birth weights according to gestational age in singleton live births for **girls** in Poland (2019)

GA at delivery [weeks]	n= 177,311	Birth weight [grams]								
		Smoothed centiles							Mean	SD
		C3	C5	C10	C50	C90	C95	C97		
23	60	421	443	472	557	652	690	721	568	91
24	76	461	491	532	650	778	829	869	646	93
25	80	492	535	591	751	920	986	1037	772	139
26	85	518	574	650	858	1072	1152	1214	861	209
27	119	555	625	719	975	1231	1325	1397	958	220
28	133	622	703	811	1109	1404	1511	1591	1095	271
29	157	724	811	930	1261	1591	1710	1800	1261	262
30	223	852	944	1069	1427	1794	1928	2028	1437	311
31	278	999	1094	1224	1611	2019	2169	2281	1596	322
32	395	1154	1253	1392	1812	2266	2432	2557	1853	366
33	632	1314	1419	1568	2025	2518	2697	2830	2041	422
34	1070	1494	1606	1764	2254	2776	2960	3095	2254	402
35	1899	1705	1820	1985	2501	3045	3231	3366	2514	446
36	3999	1935	2048	2212	2739	3296	3483	3615	2751	441
37	10,673	2200	2306	2461	2978	3531	3713	3841	2987	437
38	32,648	2473	2570	2714	3206	3739	3911	4031	3220	410
39	55,190	2642	2733	2872	3354	3878	4046	4161	3367	402
40	50,132	2757	2848	2987	3476	4003	4168	4280	3488	404
41	18,448	2845	2936	3076	3570	4095	4256	4365	3580	402
42	1014	2852	2946	3089	3595	4126	4287	4394	3597	416

Abbreviations: C—Centile, GA—gestational age, SD—standard deviation

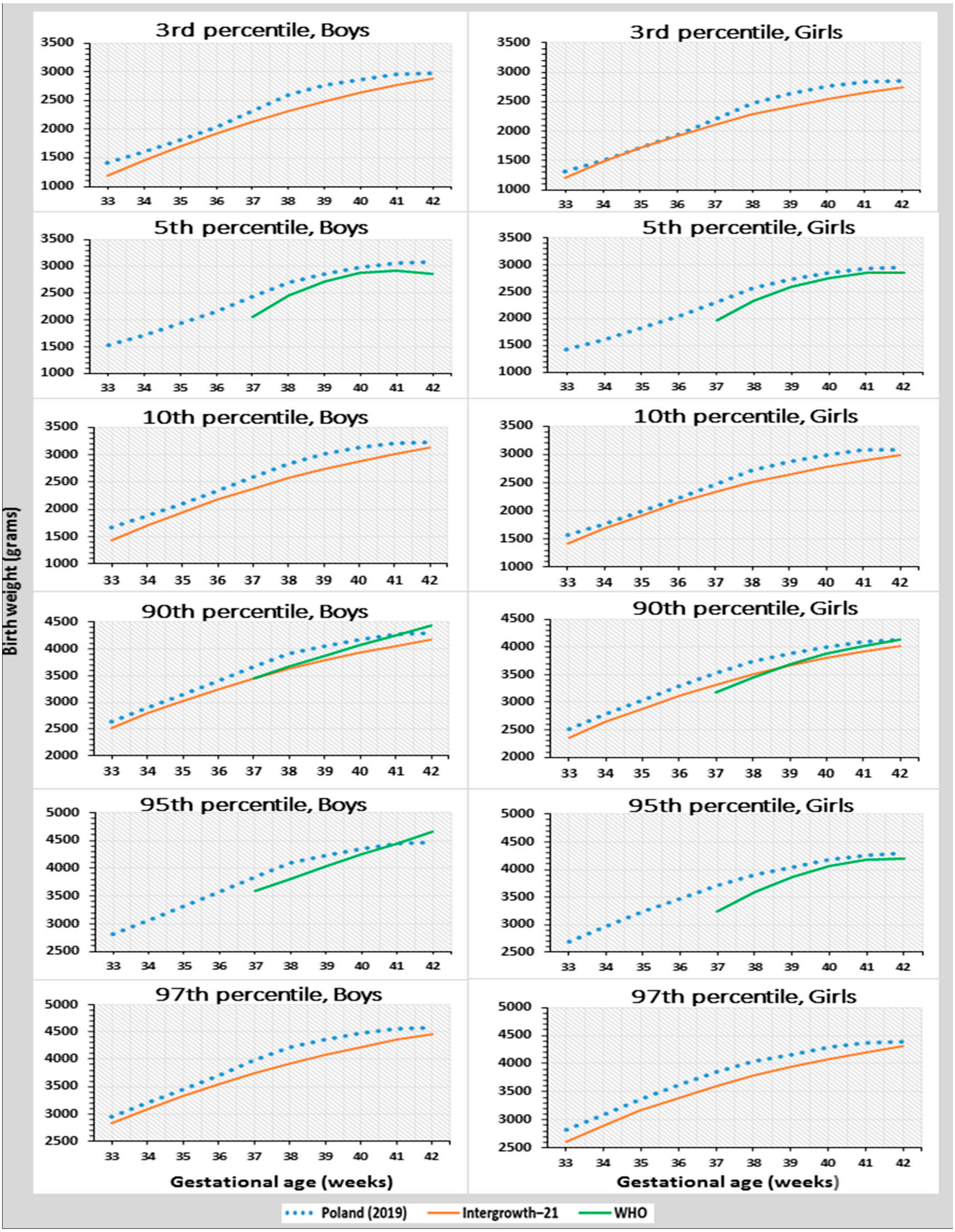

Figure S3. Birth weight at the 3rd, 5th, 10th, 90th, 95th, 97th percentiles based on gestational age among Polish newborns from singleton pregnancies, in comparison with the global references INTERGROWTH-21 and WHO
